# Development and validation of the Oxford Attitudes to Artificial Intelligence in Medicine Scale

**DOI:** 10.1016/j.jdin.2026.04.005

**Published:** 2026-04-16

**Authors:** Jonathan Kantor, Michael Morrison, Justin Ko, Yevgeniy R. Semenov, Rebecca G. Fortgang

**Affiliations:** a Department of Engineering Science, University of Oxford, Oxford, UK; b Department of Biostatistics, Epidemiology, and Informatics, University of Pennsylvania Perelman School of Medicine, Philadelphia, Pennsylvania; c Centre for Health, Law, and Emerging Technologies (HeLEx), University of Oxford, Oxford, UK; d Department of Dermatology, Stanford University, Palo Alto, California; e Department of Dermatology, Massachusetts General Hospital, Boston, Massachusetts; f Department of Psychiatry, Massachusetts General Hospital, Boston, Massachusetts

**Keywords:** artificial intelligence, confirmatory factor analysis, exploratory factor analysis, public attitudes toward medical AI, study guidelines, survey measures

## Abstract

**Objective:**

Public willingness to accept medical artificial intelligence (AI) tools affect the potential real-world impact of these evolving technologies. We therefore developed and validated the Oxford Attitudes to Artificial Intelligence in Medicine Scale (OAAIMS), an instrument designed to assess public and patient attitudes towards AI use in healthcare.

**Methods:**

Candidate domains and items were generated through literature review, expert panels, and open-ended surveys, then administered to demographically representative samples (the United Kingdom *n* = 491; the United States *n* = 500). Exploratory factor analysis using polychoric correlations was conducted on the UK sample; confirmatory factor analysis was used to test the model on the US sample. Internal consistency (McDonald’s ω), convergent validity (correlation with the attitudes toward AI scale), discriminant validity (correlation with the Oxford Vaccine Hesitancy Scale), and criterion validity (logistic regression predicting preference for human-only care) were assessed.

**Results:**

Exploratory factor analysis supported a 20-item, 4-factor structure with domains of Benefit (9 items), Trust (5 items), Task-specific Comfort (3 items), and Concerns (3 items). Confirmatory factor analysis showed good fit (root mean squared error of approximation = 0.055; standardized root mean squared residual = 0.037; comparative fit index = 0.963). Internal consistency was high for the total scale (ω = 0.95 UK, 0.96 US) and subscales (ω = 0.81-0.94). OAAIMS correlated with general AI attitudes (r = 0.70-0.71) and minimally with vaccine hesitancy (|r| ≤ 0.14). Each 1-point increase in OAAIMS score reduced the odds of preferring AI-free care by 8% to 9% (UK odds ratio of association [OR] 0.91, US OR 0.92; both *P* < .001).

**Discussion:**

The OAAIMS is a brief, psychometrically robust scale capturing 4 distinct domains of public attitudes toward medical AI, developed using demographically representative samples.

**Conclusion:**

The OAAIMS can serve as a baseline descriptor, outcome measure, or stratification variable in trials and implementation studies, permitting quantifiable tracking of patient-centered barriers and allowing for de-risking nascent technologies. OAAIMS is an informatics-ready instrument for baselining, stratifying, and longitudinally monitoring public acceptance of medical AI in trials and health-system implementations.

**Capsule Summary**

• Public willingness to accept artificial intelligence in medicine shapes real-world adoption, yet no validated scale has been developed to measure these attitudes in demographically representative general-public populations.
• The Oxford Attitudes to Artificial Intelligence in Medicine Scale offers a brief, validated instrument that clinicians and researchers can use to establish a baseline, stratify, and longitudinally track patient acceptance of artificial intelligence in medicine.

While machine learning and artificial intelligence (AI) have been used in the medical field for decades, the explosive popularity of multimodal foundation models has catapulted AI in medicine to the forefront of public consciousness.^1^, ^2^, ^3^ Despite the enthusiasm of developers, early adopters, and many clinicians, the near-term impact of AI in medicine—and particularly the pace of adoption and buy-in by patients—will also be dictated by public attitudes to and acceptance of the use of AI in health care.^4^ Thus, while the rapid pace of development and broad medical professional enthusiasm suggests that AI-based medical tools are useful, their putative clinical impact may be constrained if the public is uninterested—or even opposed—to their adoption. Indeed, as vaccine hesitancy has shown, developing medical devices and therapies is of limited use if the public remains skeptical of their utility and safety.^5^

Validated scales provide quantitative insight into attitudes and preferences that can be useful in understanding public priorities, anticipating pushback, and de-risking nascent technologies. Yet despite the broad implications of AI in medicine and the profound impact that it is already having on patient care, relatively little is known regarding public attitudes about these developments.^6^, ^7^, ^8^, ^9^, ^10^ Most existing validated measures of AI attitudes are designed to focus on AI broadly rather than medicine specifically, although some include questions regarding health care.^11^, ^12^, ^13^, ^14^, ^15^ The few existing measures that relate to attitudes about AI in medicine are focused on medical professionals’ attitudes^16^ or hospitalized patients.^17^ Instruments aimed at assessing pediatric patients,^18^ and an adult adaptation of that pediatric scale,^19^ were both developed prior to the generative AI era and do not represent ground-up scales built and validated on multinational demographically representative general public samples.

From an informatics perspective, standardized attitude measures can be embedded in predeployment assessments, used as stratification covariates in implementation trials, and linked to rollout analytics for quantifying risk acceptance. Unlike prior general AI instruments or specialty-specific tools, Oxford Attitudes to Artificial Intelligence in Medicine Scale (OAAIMS) was developed on demographically representative public samples in the United Kingdom and United States to target decisions that influence uptake in routine care. It can therefore be seen as a consumer-health informatics and implementation measure that complements performance metrics by capturing the human-factors barriers to adoption.

Although some work has explored digital health readiness more generally,^20^ quantifying public attitudes to AI in medicine provides several benefits. First, if patients and the public fear that novel developments are not in their interest, this could lead to pushback that may ultimately hamstring the development of new technologies, potentially limiting the perceived benefits of AI-based advances. Indeed, such attitudes likely affect the ways in which stakeholders, including the public, consent to data sharing, AI implementation, and broad-scale investment in AI in medicine—and indeed patients may not trust health systems to use AI appropriately.^21^^,^^22^ Second, including quantifiable endpoints can allow for more meaningful cross-study evaluation of interventions meant to either encourage the use of AI technologies in medicine or better understand the nature of enthusiasm and reluctance of patients to participate in AI-driven care, while also permitting longitudinal tracking of attitudes to AI in medicine. Third, human-factors data on AI acceptance can be used to de-risk AI-based advances by providing physician scientists, developers, and engineers with data needed to justify continued investment, as low public buy-in may increase the risk of venture failure. We therefore aimed to develop a psychometrically robust, multidimensional scale to assess public attitudes to the use of AI in medicine, the OAAIMS.

## Materials and methods

### Study population

Our general approach to developing the OAAIMS followed our earlier methodology in validated scale development.^23^, ^24^, ^25^, ^26^, ^27^ Briefly, survey instruments were developed using Qualtrics (Qualtrics International) and responses were collected through a survey panel approach using the academic survey site Prolific Academic (Oxford) using the representative sample workflow. After a thorough literature review, we conducted a pilot study on a convenience sample using both open-ended and closed-ended questions to explore domains, refine scale items, and assess the face validity of individual candidate items. To minimize topic priming, we studied “Attitudes to topics in health care.” To further minimize conditioning, we also excluded pilot participants and randomized item order.

We then developed a draft survey for use on demographically representative samples in the United Kingdom and United States. We recruited participants via Prolific Academic using quota sampling to approximate national population distributions. For the United Kingdom (exploratory factor analysis [EFA] cohort), quotas were set against the UK Office for National Statistics census distributions for age (18-24, 25-34, 35-44, 45-54, 55-64, ≥65), sex (female/male), and high-level ethnicity groups (White; Mixed/Multiple; Asian/Asian British; Black/African/Caribbean/Black British; Other). Quotas were interlocking (age × sex × race), and recruitment continued until each cell reached its target. For the United States (confirmatory factor analysis cohort), quotas were based on US Census Bureau (2015) distributions for the same age bands, sex, and US-specific race/ethnicity (Non-Hispanic White, Non-Hispanic Black, Hispanic/Latino, Non-Hispanic Asian, Other/Multiracial). Where minor imbalances arose due to attrition or screen failures, we applied poststratification weights to align sample marginals to census targets.

This work was approved by the University of Oxford Medical Sciences Interdivisional Research Ethics Committee (approval reference R81585/RE001). All procedures involving human participants were conducted in accordance with the Declaration of Helsinki and institutional policies. All participants were reimbursed less than £5/$5 for their time, provided informed consent for participation, and were permitted to withdraw at any time. Participant demographic details were self-declared and linked to responses using a unique 25-character alphanumeric identifier. Baseline demographic data included age, sex, race and ethnicity, employment status, educational background, and reported household income. Attention check items were included in the survey instrument; results were excluded from the analysis if a respondent failed 2 or more attention checks.

Qualitative analyses were performed with NVivo release 1.4 (QSR International) and statistical analyses were performed using Stata for Mac version 16 (Stata Corporation). A *P* value of .05 was considered significant for all analyses.

### Item reduction and exploratory factor analysis

We used Bartlett’s test of sphericity and the Kaiser-Meyer-Olkin measure of sampling adequacy to assess whether the data were amenable to factor analysis.^28^ We used the minimum average partial correlation for the number of principal components and a scree plot to establish the number of factors to include in the scale.^29^^,^^30^

Item reduction was performed by assessing domain relevance, examining polychoric (item-item) and polyserial (item-test) correlations, culling redundant items, and removing those with low communality or low factor loadings.^31^ In keeping with our earlier approaches to scale development, we used EFA with categorical variables and polychoric correlations to generate a factor matrix using iterated principal factor analysis followed by promax oblique factor rotation.^32^^,^^33^

### Confirmatory factor analysis

Confirmatory maximum likelihood factor analysis using structural equation modeling with a Satorra-Bentler scaled test statistic was conducted on the US sample with the model developed in the UK sample to assess robustness. Goodness-of-fit indices included the root mean squared error of approximation (RMSEA), the standardized root mean squared residual (SRMR), and the comparative fit index (CFI). Values of RMSEA ≤0.08, SRMR ≤0.08, and CFI ≥0.90 suggest acceptable fit, while values of RMSEA ≤0.05, SRMR ≤0.06, and CFI ≥0.95 suggest excellent fit.^34^

### Reliability and validity

Reliability as internal consistency was assessed using McDonald’s ω, a robust alternative to Cronbach’s alpha that does not assume equal factor loadings. This was performed for the overall OAAIMS as well as each subscale.^35^

Convergent validity was assessed by evaluating the correlation between the OAAIMS and the Attitudes Towards Artificial Intelligence (ATAI) scale, a validated 5-item scale assessing general (not medicine-specific) attitudes to AI.^36^ Concurrent criterion validity was further investigated by assessing the logistic regression odds ratios of association between OAAIMS score and participant preference for being cared for by a human-only medical team. Discriminant validity was assessed by examining the association between the OAAIMS score and the largely unrelated construct of vaccine hesitancy, as measured using the Oxford Vaccine Hesitancy Scale.

## Results

### Study population

Our sample included a total of 991 demographically representative respondents in the United Kingdom and United States; sample sizes per cohort were based on exceeding the rule of thumb regarding including 10 participants per item,^37^ assuming a maximum of 45 items for inclusion. In assessing attrition, in the UK cohort, 507 individuals initially accessed our survey; 9 failed to complete the survey (yielding a completion rate of 98.2%) and 7 (1.4%) failed at least 2 attention checks, resulting in 491 unique participants for analysis. Mean age was 47 years (95% confidence interval [CI]: 45.6, 48.3) with a range from 18 to 83. Slightly more than half of the participants were female (50.9%) and the mean household income was £49,624. In the United Kingdom, 16.1% of the participants identified as non-White, 38.9% reported having no children, and 49.6% reported working full-time.

In evaluating attrition in the US sample, 540 respondents accessed our survey; 32 failed to complete the survey (yielding a completion rate of 94.1%) and 8 (1.6%) failed at least 2 attention checks, yielding 500 unique participants. The mean age was 45.8 years (95% CI: 44.4, 47.3) with a range of 18 to 85 years. Overall, 49.8% of respondents were female and the mean household income was $92,900. In the United States, 26.7% of participants identified as non-White, 47.3% reported having no children, and 48.2% were working full-time.

Our pilot sample included 50 participants, with a mean (standard deviation) age of 38.7 years (13.7) and an age range of 18 to 67 years; 48% of respondents were female. Closed-ended and open-ended responses confirmed that all included scale items were easy to understand.

### Domains, items, and exploratory factor analysis

Based on investigator input, literature review, and open-ended responses to our pilot surveys, we considered 21 broad overlapping domains: trust, comfort, benefit, accuracy, efficiency, cost, health care access, communication, worry, concern, bias, privacy, unintended consequences, ethical challenges, quality reduction, responsibility, equity, training, awareness, empathy, and supervision. We developed a list of potential items which were iteratively winnowed to 63 items for inclusion as candidate scale items on the full sample. Of these, 43 items were removed due to redundancy, low polychoric or polyserial correlations, or low communality. The final OAAIMS included 20 items.

We performed the 20-item EFA using the UK sample; Bartlett’s test for sphericity (*P* < .0001) suggested that the data are appropriate for EFA, which was confirmed by the Kaiser-Meyer-Olkin measure of sampling adequacy, which was excellent at 0.949. Minimum average partial correlation analysis and a scree plot suggested a 4-factor solution.^38^^,^^39^

The 4 factors included in our scale included: benefit (9 items), trust (5 items), task-specific comfort (3 items), and concerns (3 items). The benefit domain reflects adoption intent and perceived utility or the potential gains that could be seen from adopting AI in medicine. The trust domain encompasses reliability and a willingness to generally incorporate AI guidance in medical decision-making. The task-specific comfort domain addresses comfort with AI involvement in specific applications such as pathology, radiology, and skin imaging analysis. The concerns domain encompasses AI risk and fear; all items in this domain were reverse scored.

This approach yielded a 4-factor 20-item scale, with possible scores ranging from 20 to 100, with higher scores representing a more open attitude to AI in medicine.

Rotated factor loadings are included in Table I, factor intercorrelations are included in Table II, and the full OAAIMS with scoring key is included in Table III.

**Table I:** Rotated factor loadings for the OAAIMS

Domain	Item	Benefit	Trust	Comfort	Concerns
Benefit	I would like AI to help me with routine health monitoring and alerts.	**0.8187**	−0.0672	0.0879	−0.0123
Benefit	I would like to receive AI-generated personalized health educational materials based on my medical records.	**0.7505**	0.0998	−0.0650	0.0005
Benefit	AI could make health care more accessible to many people.	**0.7476**	0.0644	−0.0145	0.0130
Benefit	The added efficiency of AI will reduce unnecessary procedures and visits.	**0.7326**	0.0944	−0.0134	0.0138
Benefit	I believe that AI can lead to faster development of new medical treatments.	**0.7319**	0.0380	0.0890	−0.0443
Benefit	I believe that AI can improve my understanding of medical options.	**0.7066**	0.1542	0.0731	0.0634
Benefit	AI and human health care providers could complement each other through their different strengths.	**0.6210**	−0.0482	0.2677	−0.0258
Benefit	I am hopeful that an increased use of AI in health care will free up medical professionals’ time to give patients more personalized attention.	**0.5836**	−0.0038	0.1165	−0.1506
Benefit	I am optimistic about AI’s ability to innovate and advance medical treatments.	**0.5594**	0.1499	0.1298	−0.0615
Trust	I trust AI to accurately diagnose medical conditions.	−0.0838	**0.9171**	0.0120	−0.1163
Trust	I feel that AI-based diagnostic tools are reliable.	0.0567	**0.8762**	0.0403	0.0286
Trust	I would follow a treatment plan recommended by AI.	0.1404	**0.7984**	−0.0862	−0.0395
Trust	I trust AI-enabled telemedicine platforms to provide correct diagnoses and treatment plans.	0.1156	**0.6896**	0.1664	0.0003
Trust	I believe AI tools are at least as accurate as a human medical provider.	0.1011	**0.6379**	0.0423	−0.0392
Comfort	I am comfortable with AI helping doctors interpret pathology slides from a biopsy and generate a diagnosis.	0.0668	0.0808	**0.8793**	0.0189
Comfort	I am comfortable with AI helping doctors interpret radiology scans such as x-rays.	0.0798	−0.0213	**0.8195**	−0.0370
Comfort	I am comfortable with AI helping doctors evaluate photographs of my skin lesions.	0.1886	0.2644	**0.4679**	−0.0185
Concerns	I feel anxious about AI making decisions in health care.	0.0962	−0.1722	−0.1121	**0.7842**
Concerns	I fear that increased use of AI in health care will lead to less human contact in medical services.	−0.1284	0.0279	0.1460	**0.7637**
Concerns	I am concerned about the long-term effects of AI on the quality of health care.	−0.0479	−0.0835	−0.0686	**0.6783**

The bold values indicate primary factor loadings.

*AI*, Artificial intelligence; *OAAIMS*, Oxford Attitudes to Artificial Intelligence in Medicine Scale.

**Table II:** Factor intercorrelations

Factor	Benefit	Trust	Comfort	Concerns
Benefit	1			
Trust	0.6638	1		
Comfort	0.673	0.606	1	
Concerns	0.5745	0.5915	0.4519	1

**Table III:** The OAAIMS

#	Item	Domain
1	I would like AI to help me with routine health monitoring and alerts.	Benefit
2	I would like to receive AI-generated personalized health educational materials based on my medical records.	Benefit
3	AI could make health care more accessible to many people.	Benefit
4	The added efficiency of AI will reduce unnecessary procedures and visits.	Benefit
5	I believe that AI can lead to faster development of new medical treatments.	Benefit
6	I believe that AI can improve my understanding of medical options.	Benefit
7	AI and human health care providers could complement each other through their different strengths.	Benefit
8	I am hopeful that an increased use of AI in health care will free up medical professionals’ time to give patients more personalized attention.	Benefit
9	I am optimistic about AI’s ability to innovate and advance medical treatments.	Benefit
10	I trust AI to accurately diagnose medical conditions.	Trust
11	I feel that AI-based diagnostic tools are reliable.	Trust
12	I would follow a treatment plan recommended by AI.	Trust
13	I trust AI-enabled telemedicine platforms to provide correct diagnoses and treatment plans.	Trust
14	I believe AI tools are at least as accurate as a human medical provider.	Trust
15	I am comfortable with AI helping doctors interpret pathology slides from a biopsy and generate a diagnosis.	Comfort
16	I am comfortable with AI helping doctors interpret radiology scans such as x-rays.	Comfort
17	I am comfortable with AI helping doctors evaluate photographs of my skin lesions.	Comfort
18	I feel anxious about AI making decisions in health care.	Concerns
19	I fear that increased use of AI in health care will lead to less human contact in medical services.	Concerns
20	I am concerned about the long-term effects of AI on the quality of health care.	Concerns

All items are scored using a 5-point Likert scale ranging from disagree completely to agree completely, including a neutral option (neither agree nor disagree). Items 18 to 20 are reverse scored. The total score ranges from 20 to 100, with higher scores reflecting a more favorable attitude to AI in medicine.

*AI*, Artificial intelligence; *OAAIMS*, Oxford Attitudes to Artificial Intelligence in Medicine Scale.

### Confirmatory factor analysis

To assess the model developed in the EFA, a CFA was performed on the US sample. The confirmatory model created with structural equation modeling and a Satorra-Bentler scaled test statistic of the 4-factor 20-item model demonstrated adequate goodness-of-fit characteristics, with RMSEA = 0.055, SRMR = 0.037, and CFI = 0.963.

The mean (95% CI) OAAIMS score was 65.12 (63.76, 66.47) in the UK sample and 61.85 (60.30, 63.39) in the US sample (*P* = .0018). Descriptive statistics for each subscale in each region are shown in Table IV.

**Table IV:** Descriptive statistics and internal consistency (McDonald’s ω) for OAAIMS domains: UK vs US, with *P* values and Cohen’s d

Domain/scale	UK mean (95% CI)	US mean (95% CI)	*P* value	Cohen’s d	ω (UK)	ω (US)
Overall OAAIMS	65.12 (63.76-66.47)	61.85 (60.30-63.39)	.002	0.20	0.95	0.96
Benefit	32.47 (31.84-33.10)	31.26 (30.54-31.98)	.014	0.16	0.92	0.93
Trust	14.79 (14.38-15.20)	13.80 (13.33-14.27)	.002	0.20	0.92	0.94
Comfort	10.76 (10.49-11.02)	10.06 (9.77-10.35)	<.001	0.22	0.88	0.88
Concerns	7.08 (6.83-7.33)	6.76 (6.49-7.03)	.088	0.11	0.81	0.84

*P* values are based on *t* tests, assessing the statistical significance of differences in the distributions between the 2 independent samples.

*AI*, Artificial intelligence; *CI*, confidence interval; *OAAIMS*, Oxford Attitudes to Artificial Intelligence in Medicine Scale.

### Reliability and validity

Reliability as internal consistency was assessed using McDonald’s ω. Values for the overall scale were very good at 0.95 and 0.96 in the United Kingdom and United States, respectively. Omega values for the 4 subscales are included in Table IV.

Convergent validity between the OAAIMS score and the ATAI scale demonstrated a correlation coefficient of 0.70 (*P* < .0001) in the United Kingdom and 0.71 (*P* < .0001) in the United States, further bolstering our validity argument.^40^ Concurrent criterion validity was further highlighted by the association between the OAAIMS score and preference for a human-only medical team over 1 that uses AI. For each one-point increase in OAAIMS score, the logistic regression odds ratios of association between this increase and the odds of preferring a human-only medical team was 0.91 (0.89, 0.93), *P* < .0001 in the UK sample and 0.92 (0.90, 0.93), *P* < .0001 for the US sample. Thus, for each one-point increase in OAAIMS score (which ranges from 20-100, with higher scores suggesting a more positive attitude to AI in medicine), respondents were 8% to 9% less likely to state that they prefer being cared for by a medical team that does not use AI. This finding highlights the clinical utility of the OAAIMS in predicting an actionable and clinically meaningful preference. Together, these findings support the construct validity of the OAAIMS by combining theoretical association (with the ATAI) and clinical decision-making.

Discriminant validity was demonstrated by evaluating the correlation between an unrelated concept, vaccine hesitancy, assessed using the Oxford Vaccine Hesitancy Scale, and the OAAIMS score. The correlation coefficients were −0.14 in the United Kingdom and 0.03 in the United States, implying minimal shared variance (< 2%) and highlighting that the OAAIMS measures a concept distinct from the general technoskepticism or mistrust that may underlie vaccine hesitancy.

## Discussion

This study demonstrated the development and validation of the OAAIMS, a multidimensional benchmark scale to assess public attitudes to the use of AI in medicine, across demographically representative populations in the United Kingdom and United States. The scale ultimately included 4 domains: benefit, trust, task-specific comfort, and concerns. While these factors are correlated, with coefficients ranging from 0.45 to 0.67 (Table II), the factor structure suggests that each domain captures unique variance. That is, each dimension reflects a distinct stance toward AI in medicine: a perception of future-oriented system-level benefit is different from the trust needed to delegate decision-making using currently available technology or the granular comfort associated with specific tasks, while concerns regarding the role of AI in medicine do not simply reflect an absence of benefit perception, trust, or task-specific comfort.

OAAIMS scores were higher in the United Kingdom than in the United States, suggesting a slightly more positive attitude to AI in medicine in our UK sample (Table IV). Given the small absolute magnitude of the difference (less than 1.5 points per subscale and less than 4 points for the overall score), these statistically significant differences likely reflect the large sample size. Indeed, this is confirmed by the small effect size (Cohen’s d = 0.20); while a one-fifth standard deviation difference between scores in the United States and United Kingdom is likely not clinically meaningful, future studies evaluating regional differences in AI in medicine attitudes may be valuable.

The practical clinical utility of the OAAIMS is highlighted by our validation data on preferences for a human-only medical team; given the association between increasing OAAIMS score and decreasing odds of preferring AI-free medical care, future studies could explore appropriate cutoffs to better understand what population-level or patient-level OAAIMS scores are associated with clinically meaningful medical AI hesitancy or refusal, and what interventions, system modifications, or risk factors are associated with these sentiments. Determining an appropriate gold standard for such studies may be an important first step.

This study has several strengths. First, we used an iterative approach, layering expert opinion, literature review, and open-ended survey data to explore a wide range of possible domains and items for inclusion, following an established framework for developing and validating scales. Second, we relied on large demographically representative samples in 2 countries with a shared language but with cultural differences and different health care systems—and the robustness of our scale was highlighted by the similar score distributions, and by results of the CFA demonstrating good model fit in the US sample for a factor model derived in the UK sample. Third, we adopted a rigorous approach using polychoric correlation matrices when analyzing Likert data (a computationally intensive but statistically conservative approach) and saw a clean factor structure in the EFA and adequate goodness-of-fit statistics in the CFA, underscoring the robustness of our model and its psychometric rigor.^41^^,^^42^ Fourth, both the validity and potential clinical utility of our scale were highlighted by the association between a preference for human-only medical care and OAAIMS scores, with an 8% to 9% reduction in the odds of preferring such care for each one-point increase in OAAIMS score. Moreover, convergent validity with the ATAI scale and our high reliability as measured by internal consistency further support the validity and reliability of this novel measure. Fifth, the simple 4-domain structure is intuitive and amenable to use for describing baseline data, as an outcome measure in AI in medicine implementation studies, or for tracking attitudinal shifts over time in a rapidly changing landscape of AI applications in medicine. The scale is highly feasible to administer, as it contains only 20 items and takes less than 4 minutes to complete, again underscoring its potential clinical utility.

Our work also has several important limitations. First, participants were recruited through an academic survey panel company. While this offers many benefits, including access to demographically representative samples, these platforms also introduce the risk of multiple biases, including volunteer and digital-access biases. Still, all clinical studies share some of this risk, and this is partially mitigated by the large size and demographic representativeness of the samples, although the ways in which these populations differ from other specialized populations, including those more likely to be seeking medical care, is an area for future investigation. Second, this was a cross-sectional study and relied on self-report. We do not yet know whether self-reported AI attitudes will remain stable over time or predict future behavioral decisions, although future work may investigate these open questions. Third, while we investigated samples from 2 populations, broader cross-cultural work will be important for external validation of this scale and may improve generalizability and allow for broader implementation. While 1 factor includes 3 items assessing specific implementations of AI in health care—largely assisting clinicians in interpreting visual inputs—other specific implementations may merit further iterations of the scale. Fourth, investigating appropriate cutoffs, after defining questions, a gold standard outcome of interest, may be helpful in dividing respondents into those that are “medical AI hesitant” and those who are not. Future work in defining such outcomes is warranted. Fifth, OAAIMS items predominantly frame AI as a decision-support tool that assists clinicians, reflecting currently dominant deployments (and the use cases most familiar to both patients and clinicians). The rapidly emerging paradigm of agentic AI, in which autonomous systems plan, reason, and execute multistep clinical tasks with reduced human oversight, may elicit qualitatively different attitudes regarding trust, comfort, and concern. Public reactions to autonomous agents acting on patient data, coordinating care, or triage, with or without clinician-in-the-loop confirmation, may diverge meaningfully from those captured here, and future iterations of the scale may need to incorporate additional items addressing these agentic approaches.

## Conclusions

As studies, clinical efforts, and industry ventures continue to expand the use of AI in medicine at a seemingly exponential rate, the need for a quantitative benchmark for studying patient and public receptivity to these interventions increases. Funding agencies, investors, and the public at large expect meaningful and trackable data to assess whether interventions will meet thresholds for acceptance, and developing a baseline validated scale for these attitudes is an important first step. The OAAIMS, with its clean factor structure assessing 4 domains, could serve as a useful tool for clinicians and researchers—from those developing AI-assisted technologies in health care to clinicians caring for patients. The observed association between higher OAAIMS scores and lower odds of preferring AI-free care suggests the scale captures a decision-salient construct; informatics teams can exploit this link to set acceptance thresholds, prospectively identify groups at risk for refusal, and evaluate targeted engagement strategies. Ultimately, the OAAIMS could be used as a stratification or modification variable in clinical trials evaluating everything from AI-enabled diagnostics to personalized treatment approaches. Moreover, as AI deployment in medicine evolves beyond first-order substitution and towards integrated, autonomous, and agentic workflows, baseline measurement of public attitudes provides a foundation for tracking how public acceptance shifts as the deployment paradigm changes. Use of the OAAIMS may help provide a uniform quantifiable metric of attitudes to AI in medicine that could have broad applicability and ultimately allow clinicians and researchers to better assess their patients’ priorities and preferences—prerequisites to the deployment of AI-augmented approaches that could improve the health of patients more broadly.

## Conflicts of interest

None disclosed.

## References

[1] Moor M., Banerjee O., Abad Z.S.H. (2023). Foundation models for generalist medical artificial intelligence. Nature.

[2] Yan S., Yu Z., Primiero C. (2025). A multimodal vision foundation model for clinical dermatology. Nat Med.

[3] Thirunavukarasu A.J., Ting D.S.J., Elangovan K., Gutierrez L., Tan T.F., Ting D.S.W. (2023). Large language models in medicine. Nat Med.

[4] Witkowski K., Dougherty R.B., Neely S.R. (2024). Public perceptions of artificial intelligence in healthcare: ethical concerns and opportunities for patient-centered care. BMC Med Ethics.

[5] Larson H.J., Gakidou E., Murray C.J.L., Longo D.L. (2022). The vaccine-hesitant moment. N Engl J Med.

[6] Moy S., Irannejad M., Jeanneret Manning S. (2024). Patient perspectives on the use of artificial intelligence in health care: a scoping review. J Patient Cent Res Rev.

[7] Beets B., Newman T.P., Howell E.L., Bao L., Yang S. (2023). Surveying public perceptions of artificial intelligence in health care in the United States: systematic review. J Med Internet Res.

[8] Steerling E., Siira E., Nilsen P., Svedberg P., Nygren J. (2023). Implementing AI in healthcare—the relevance of trust: a scoping review. Front Health Serv.

[9] Giddings R.M., Joseph A.B., Callender T.M. (2024). Factors influencing clinician and patient interaction with machine learning-based risk prediction models: a systematic review. Lancet Digit Health.

[10] Young A.T., Amara D., Bhattacharya A., Wei M.L. (2021). Patient and general public attitudes towards clinical artificial intelligence: a mixed methods systematic review. Lancet Digital Health.

[11] Schepman A., Rodway P. (2020). Initial validation of the general attitudes towards Artificial Intelligence Scale. Comput Hum Behav Rep.

[12] Koverola M., Kunnari A., Sundvall J., Laakasuo M. (2022). General attitudes towards robots scale (GAToRS): a new instrument for social surveys. Int J Soc Robot.

[13] Park J., Woo S.E., Kim J. (2024). Attitudes towards artificial intelligence at work: scale development and validation. J Occup Organ Psychol.

[14] Grassini S. (2023). Development and validation of the AI attitude scale (AIAS-4): a brief measure of general attitude toward artificial intelligence. Front Psychol.

[15] Békés V., Bőthe B., Aafjes-van Doorn K. (2025). Acceptance of using artificial intelligence and digital technology for mental health interventions: the development and initial validation of the UTAUT-AI-DMHI. Clin Psychol Psychother.

[16] Laupichler M.C., Aster A., Meyerheim M., Raupach T., Mergen M. (2024). Medical students' AI literacy and attitudes towards AI: a cross-sectional two-center study using pre-validated assessment instruments. BMC Med Educ.

[17] Busch F., Hoffmann L., Xu L. (2025). Multinational attitudes toward AI in health care and diagnostics among hospital patients. JAMA Netw Open.

[18] Sisk B.A., Antes A.L., Burrous S., DuBois J.M. (2020). Parental attitudes toward artificial intelligence-driven precision medicine technologies in pediatric healthcare. Children (Basel).

[19] Sisk B.A., Antes A.L., Lin S.C., Nong P., DuBois J.M. (2025). Validating a novel measure for assessing patient openness and concerns about using artificial intelligence in healthcare. Learn Health Syst.

[20] Rising K.L., Guth A., Gentsch A.T. (2024). Development and preliminary validation of a screener for digital health readiness. JAMA Netw Open.

[21] Nong P., Platt J. (2025). Patients' trust in health systems to use artificial intelligence. JAMA Netw Open.

[22] Shevtsova D., Ahmed A., Boot I.W.A. (2024). Trust in and acceptance of artificial intelligence applications in medicine: mixed methods study. JMIR Hum Factors.

[23] Kantor J., Vanderslott S., Morrison M., Pollard A.J., Carlisle R.C. (2023). The Oxford Needle Experience (ONE) scale: a UK-based and US-based online mixed-methods psychometric development and validation study of an instrument to assess needle fear, attitudes and expectations in the general public. BMJ Open.

[24] Kantor B.N., Kantor J. (2021). Development and validation of the Oxford Pandemic Attitude Scale-COVID-19 (OPAS-C): an internet-based cross-sectional study in the UK and USA. BMJ Open.

[25] Kantor J., Carlisle R.C., Morrison M., Pollard A.J., Vanderslott S. (2024). Oxford Vaccine Hesitancy Scale (OVHS): a UK-based and US-based online mixed-methods psychometric development and validation study of an instrument to assess vaccine hesitancy. BMJ Open.

[26] Kantor J., Carlisle R.C., Vanderslott S., Pollard A.J., Morrison M. (2025). Development and validation of the Oxford Benchmark Scale for Rating Vaccine Technologies (OBSRVT), a scale for assessing public attitudes to next-generation vaccine delivery technologies. Hum Vaccin Immunother.

[27] Kantor J., Aasi S.Z., Alam M., Paoli J., Ratner D. (2024). Development and validation of the Oxford skin cancer treatment scale, a patient-reported outcome measure for health-related quality of life and treatment satisfaction after skin cancer treatment. Dermatol Surg.

[28] Lorenzo-Seva U., Ferrando P.J. (2021). MSA: the forgotten index for identifying inappropriate items before computing exploratory item factor analysis. Methodology.

[29] Horn J.L. (1965). A rationale and test for the number of factors in factor analysis. Psychometrika.

[30] Watkins M.W. (2022).

[31] Cappelleri J.C., Lundy J.J., Hays R.D. (2014). Overview of classical test theory and item response theory for the quantitative assessment of items in developing patient-reported outcomes measures comment. Clin Ther.

[32] Holgado–Tello F.P., Chacón–Moscoso S., Barbero–García I., Vila–Abad E. (2010). Polychoric versus Pearson correlations in exploratory and confirmatory factor analysis of ordinal variables. Qual Quant.

[33] Flora D.B., Curran P.J. (2004). An empirical evaluation of alternative methods of estimation for confirmatory factor analysis with ordinal data. Psychol Methods.

[34] Browne M.W., Cudeck R. (1992). Alternative ways of assessing model fit. Sociol Methods Res.

[35] Streiner D.L. (2003). Starting at the beginning: an introduction to coefficient alpha and internal consistency. J Personal Assess.

[36] Sindermann C., Sha P., Zhou M. (2021). Assessing the attitude towards artificial intelligence: introduction of a short measure in German, Chinese, and English language. KI - Künstl Intell.

[37] Mundfrom D.J., Shaw D.G., Ke T.L. (2005). Minimum sample size recommendations for conducting factor analyses. Int J Test.

[38] Garrido L.E., Abad F.J., Ponsoda V. (2011). Performance of velicer's minimum average partial factor retention method with categorical variables. Educ Psychol Meas.

[39] Ledesma R.D., Valero-Mora P., Macbeth G. (2015). The scree test and the number of factors: a dynamic graphics approach. Span J Psychol.

[40] Betsch C., Schmid P., Heinemeier D., Korn L., Holtmann C., Bohm R. (2018). Beyond confidence: development of a measure assessing the 5C psychological antecedents of vaccination. PLoS one.

[41] DeWees T.A., Mazza G.L., Golafshar M.A., Dueck A.C. (2020). Investigation into the effects of using normal distribution theory methodology for likert scale patient-reported outcome data from varying underlying distributions including floor/ceiling effects. Value Health.

[42] Wu H., Leung S.-O. (2017). Can likert scales be treated as interval scales?—A simulation study. J Social Serv Res.

